# Supplementary material for: Modular safe-harbor transgene insertion for targeted single-copy and extrachromosomal array integration in Caenorhabditis elegans
Source: G3 (Bethesda). 2022 Jul 28;12(9):jkac184. doi: 10.1093/g3journal/jkac184 (PMC9434227; doi:10.1093/g3journal/jkac184)
Supplement: jkac184_Figure_S1 [file jkac184_figure_s1.pdf]

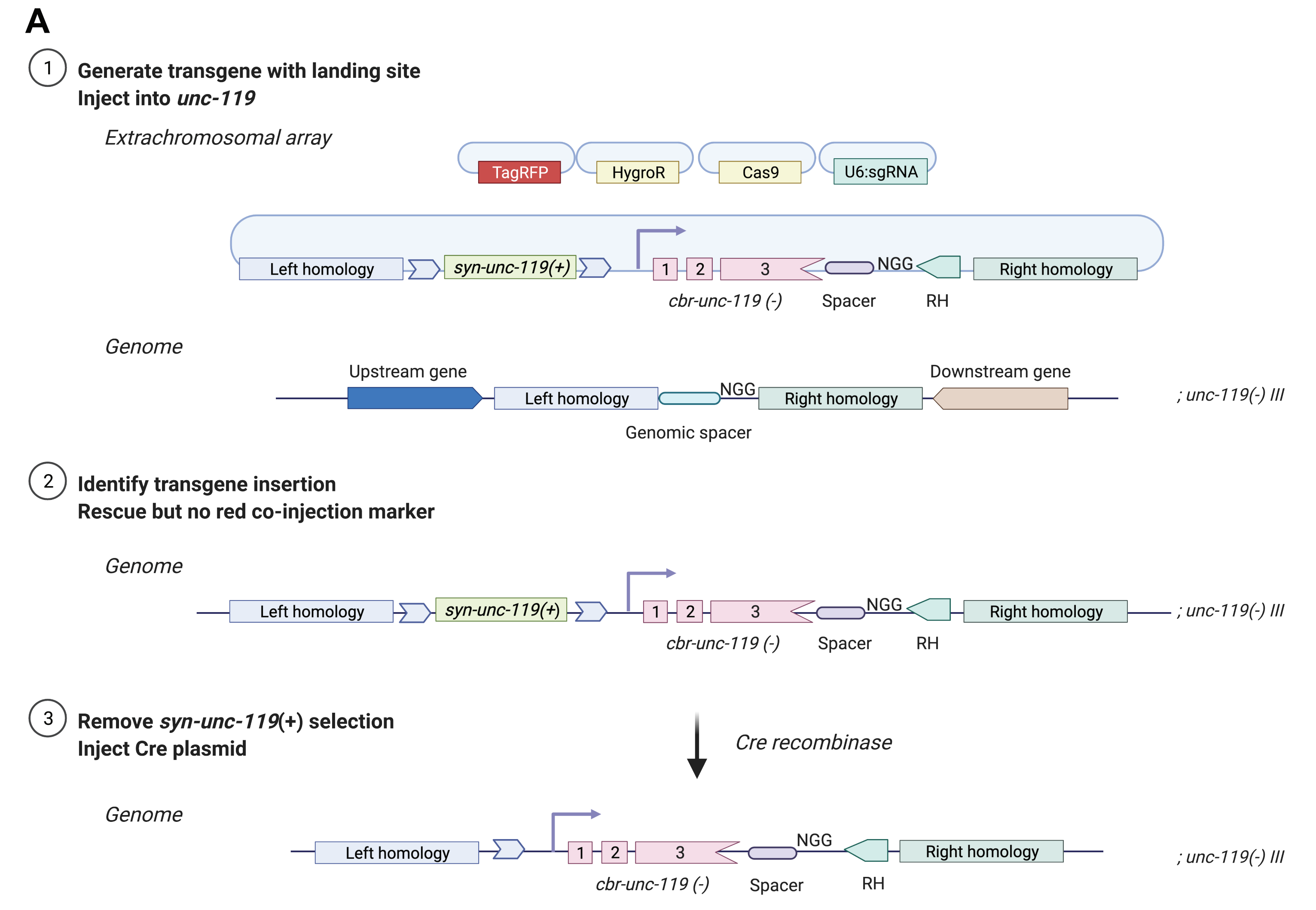
A
1
Generate transgene with landing site
Inject into unc-119
Extrachromosomal array
TagRFP
HygroR
Cas9
U6:sgRNA
Left homology
syn-unc-119(+)
1
2
3
NGG
Right homology
cbr-unc-119 (-)
Spacer
RH
Genome
Upstream gene
Downstream gene
Left homology
NGG
Right homology
; unc-119(-) III
Genomic spacer
2
Identify transgene insertion
Rescue but no red co-injection marker
Genome
Left homology
syn-unc-119(+)
1
2
3
NGG
Right homology
; unc-119(-) III
cbr-unc-119 (-)
Spacer
RH
3
Remove syn-unc-119(+) selection
Inject Cre plasmid
Cre recombinase
Genome
Left homology
1
2
3
NGG
Right homology
; unc-119(-) III
cbr-unc-119 (-)
Spacer
RH
