# Supplementary material for: Modular safe-harbor transgene insertion for targeted single-copy and extrachromosomal array integration in Caenorhabditis elegans
Source: G3 (Bethesda). 2022 Jul 28;12(9):jkac184. doi: 10.1093/g3journal/jkac184 (PMC9434227; doi:10.1093/g3journal/jkac184)
Supplement: jkac184_Figure_S2 [file jkac184_figure_s2.pdf]

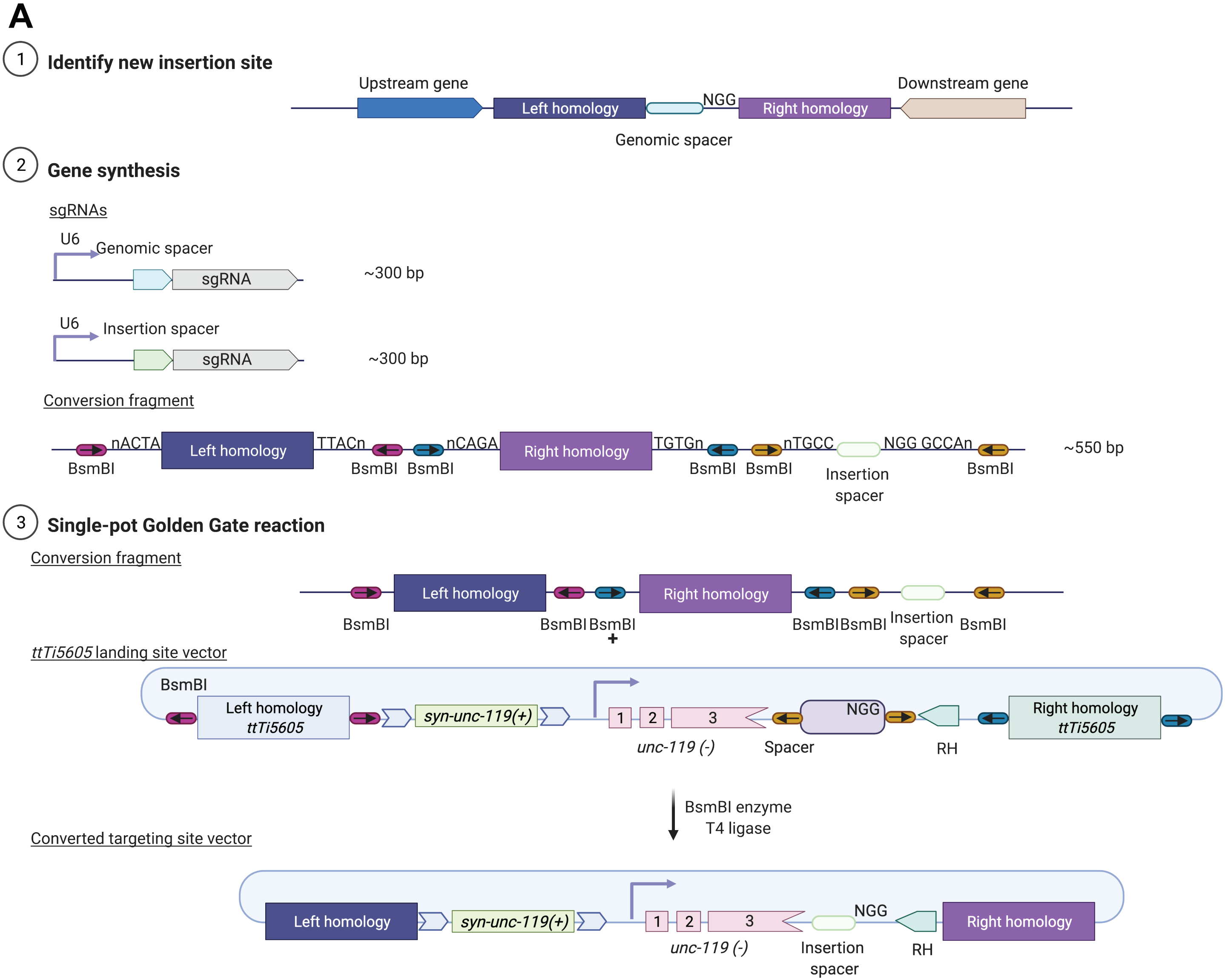
A
1 Identify new insertion site
Upstream gene
Left homology
NGG
Right homology
Downstream gene
Genomic spacer
2 Gene synthesis
sgRNAs
U6
Genomic spacer
sgRNA
~300 bp
U6
Insertion spacer
sgRNA
~300 bp
Conversion fragment
nACTA
Left homology
TTACn
nCAGA
Right homology
TGTGn
nTGCC
NGG GCCAn
~550 bp
BsmBI
BsmBI BsmBI
BsmBI BsmBI
Insertion spacer
BsmBI
3 Single-pot Golden Gate reaction
Conversion fragment
Left homology
Right homology
BsmBI
BsmBI BsmBI
BsmBI BsmBI
Insertion spacer
BsmBI
+
ttTi5605 landing site vector
BsmBI
Left homology ttTi5605
syn-unc-119(+)
1 2 3
unc-119 (-)
NGG
Spacer
RH
Right homology ttTi5605
BsmBI enzyme
T4 ligase
Converted targeting site vector
Left homology
syn-unc-119(+)
1 2 3
unc-119 (-)
Insertion spacer
NGG
RH
Right homology
