# Supplementary material for: Modular safe-harbor transgene insertion for targeted single-copy and extrachromosomal array integration in Caenorhabditis elegans
Source: G3 (Bethesda). 2022 Jul 28;12(9):jkac184. doi: 10.1093/g3journal/jkac184 (PMC9434227; doi:10.1093/g3journal/jkac184)

MosTI (Modular single-copy and Transgene Insertion)

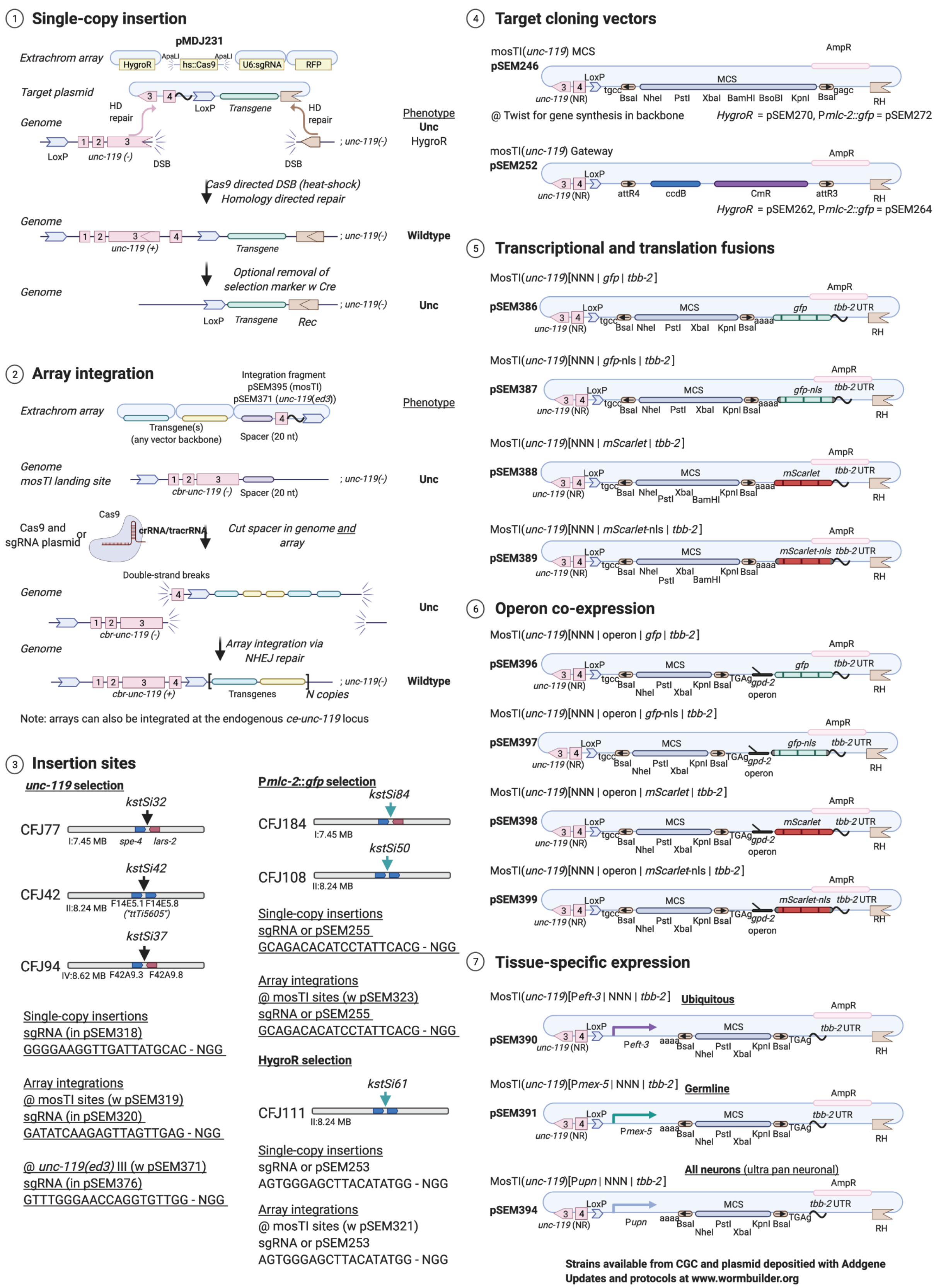

Supplement: jkac184_Figure_S3 [file jkac184_figure_s3.pdf]
