# Supplementary figures and images for: Modular safe-harbor transgene insertion for targeted single-copy and extrachromosomal array integration in Caenorhabditis elegans
Source: G3 (Bethesda). 2022 Jul 28;12(9):jkac184. doi: 10.1093/g3journal/jkac184 (PMC9434227; doi:10.1093/g3journal/jkac184)

**A**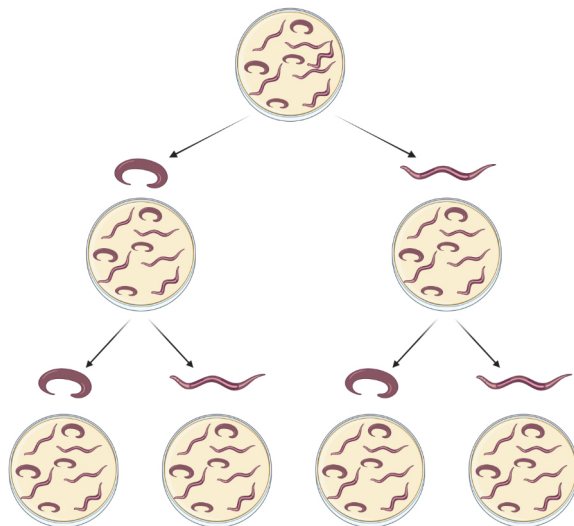**B**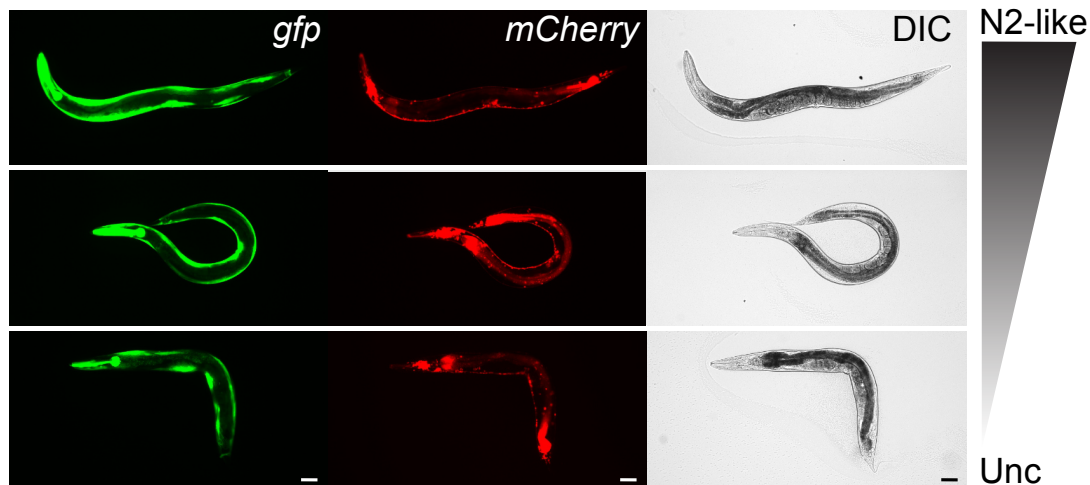

Supplement: jkac184_Figure_S4 [file jkac184_figure_s4.pdf]

**A**

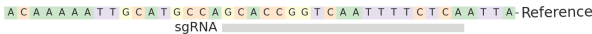

## CFJ154

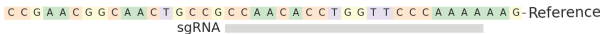

## CFJ155

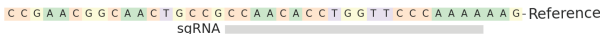

## CFJ156

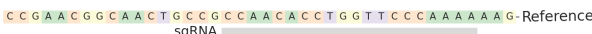

# B

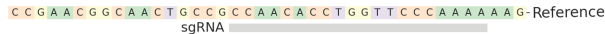

## CFJ158

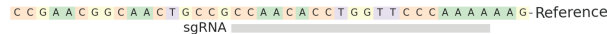

## CFJ159

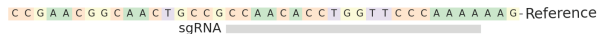

## CFJ160

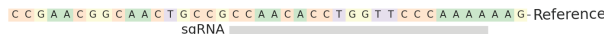

Supplement: jkac184_Figure_S5 [file jkac184_figure_s5.pdf]

A

GFP expression

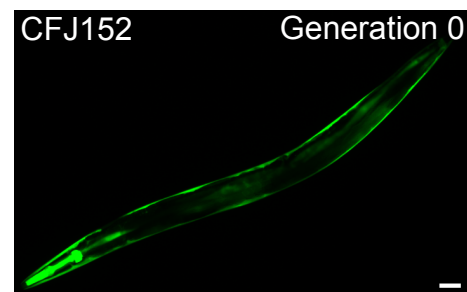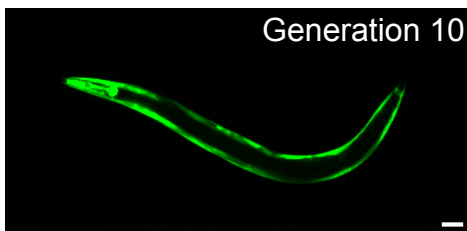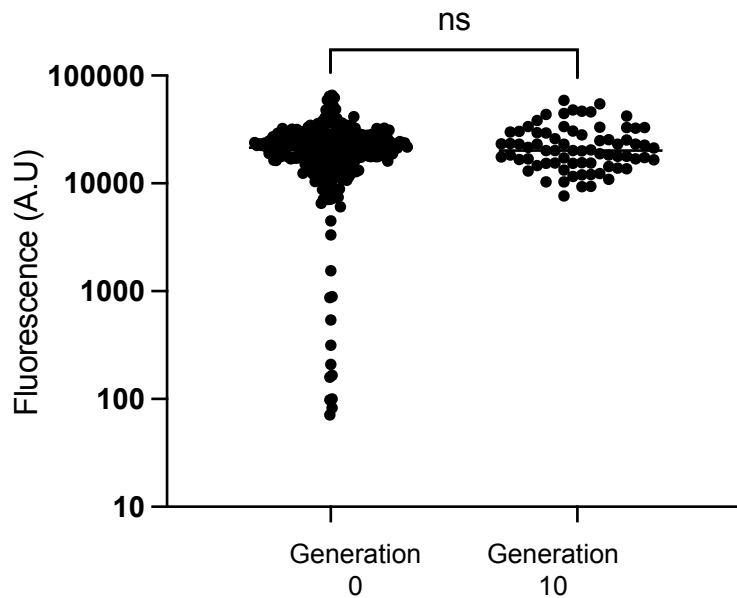

B

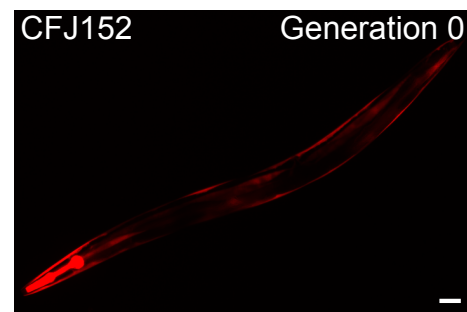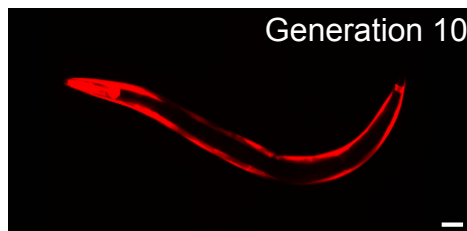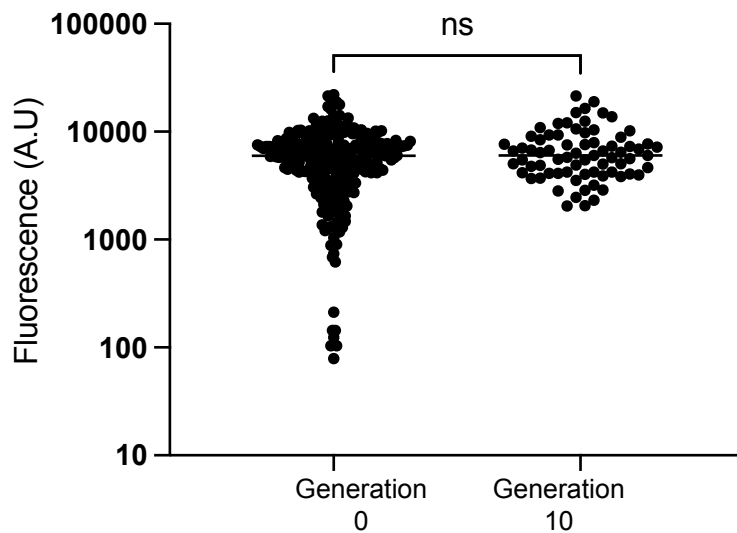

Supplement: jkac184_Figure_S6 [file jkac184_figure_s6.pdf]

**A**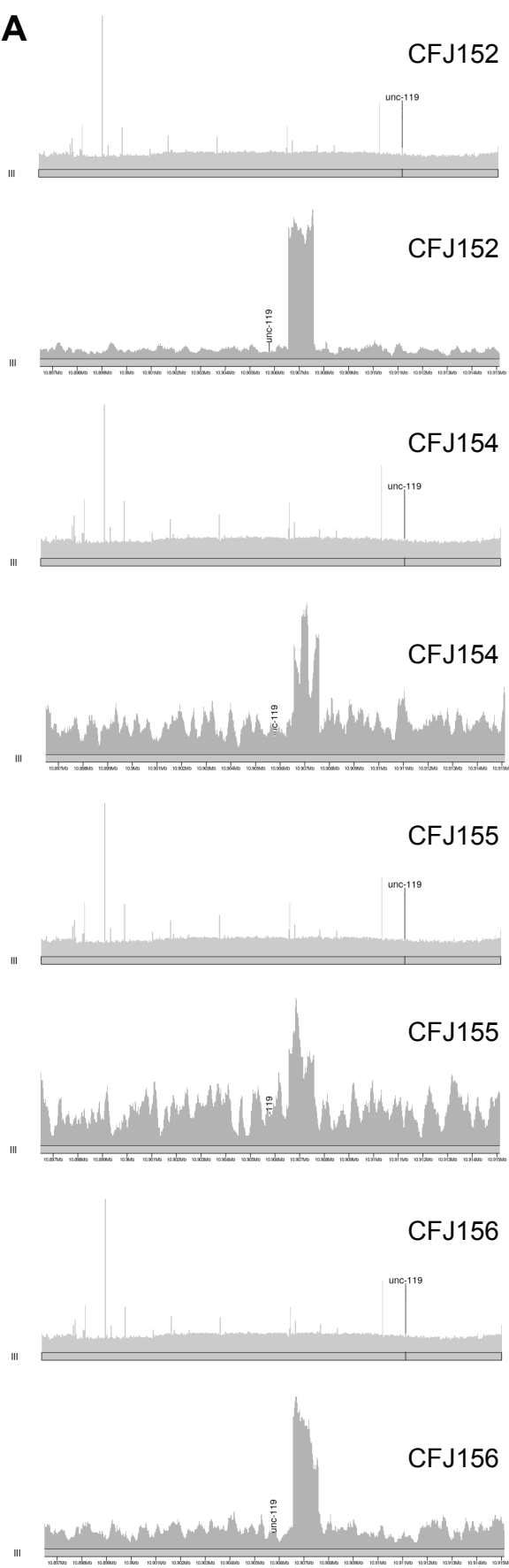**B**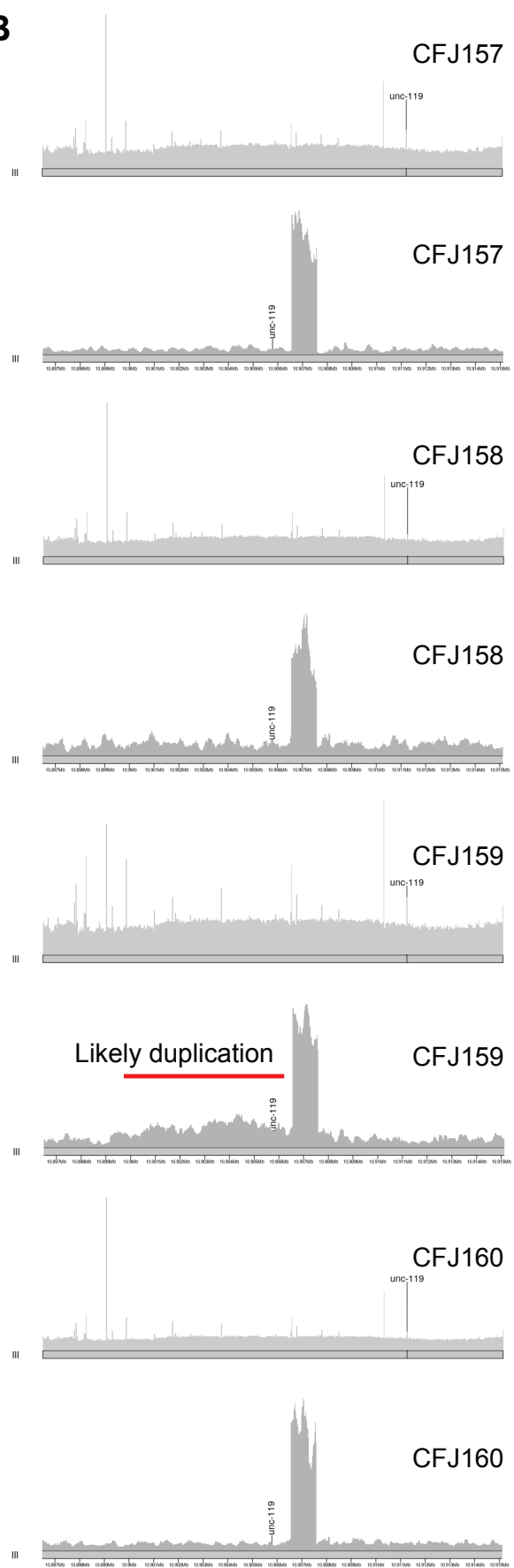

Supplement: jkac184_Figure_S7 [file jkac184_figure_s7.pdf]
