# Supplementary material for: Modular safe-harbor transgene insertion for targeted single-copy and extrachromosomal array integration in Caenorhabditis elegans
Source: G3 (Bethesda). 2022 Jul 28;12(9):jkac184. doi: 10.1093/g3journal/jkac184 (PMC9434227; doi:10.1093/g3journal/jkac184)
Supplement: jkac184_Supplemental_Material_Legends [file jkac184_supplemental_material_legends.docx]

### Supplementary Materials

**Figure S1** - Two-step protocol to generate initial MosTI landing sites

**Figure S2** - Generating new MosTI landing site.

**Figure S3** - MosTI overview.

**Figure S4** - Position effect variegation following array integration.

**Figure S5** - Indels in integration fragments within integrated arrays.

**Figure S6** - Fluorophore expression is unchanged after ten generations for integrated array.

**Figure S7** - Sequencing read coverage to detect large-scale duplications and deletions .

**Table S1** - Strains, plasmids, insertion sites, SNPs CFJ42 and CFJ77

**File S1** - GenBank annotated plasmid sequence files.
